# Curation of cancer hallmark-based genes and pathways for *in silico* characterization of chemical carcinogenesis

**DOI:** 10.1093/database/baaa045

**Published:** 2020-06-15

**Authors:** Peir-In Liang, Chia-Chi Wang, Hsien-Jen Cheng, Shan-Shan Wang, Ying-Chi Lin, Pinpin Lin, Chun-Wei Tung

**Affiliations:** 1 Phd Program in Toxicology, Kaohsiung Medical University, 100 Shiquan 1st Road, Kaohsiung 80706, Taiwan; 2 Department of Pathology, Kaohsiung Medical University Hospital, Kaohsiung Medical University, 100 Ziyou 1st Road, Kaohsiung 80706, Taiwan; 3 Department and Graduate Institute of Veterinary Medicine, School of Veterinary Medicine, National Taiwan University, 1 Section 4 Roosevelt Rd, Taipei 10617, Taiwan; 4 National Institute of Environmental Health Sciences, National Health Research Institutes, 35 Keyan Road, Miaoli County 35053, Taiwan; 5 Graduate Institute of Data Science, College of Management, Taipei Medical University, 250 Wuxing Street, Taipei 10675, Taiwan; 6 School of Pharmacy, Kaohsiung Medical University, 100 Shiquan 1st Road, Kaohsiung 80706, Taiwan

**Keywords:** cancer hallmark, curation, the Halifax Project

## Abstract

Exposure to toxic substances in the environment is one of the most important causes of cancer. However, the time-consuming process for the identification and characterization of carcinogens is not applicable to a huge amount of testing chemicals. The data gaps make the carcinogenic risk uncontrollable. An efficient and effective way of prioritizing chemicals of carcinogenic concern with interpretable mechanism information is highly desirable. This study presents a curation work for genes and pathways associated with 11 hallmarks of cancer (HOCs) reported by the Halifax Project. To demonstrate the usefulness of the curated HOC data, the interacting HOC genes and affected HOC pathways of chemicals of the three carcinogen lists from IARC, NTP and EPA were analyzed using the *in silico* toxicogenomics ChemDIS system. Results showed that a higher number of affected HOCs were observed for known carcinogens than the other chemicals. The curated HOC data is expected to be useful for prioritizing chemicals of carcinogenic concern.

Database URL: The HOC database is available at https://github.com/hocdb-KMU-TMU/hocdb and the website of Database journal as Supplementary Data.

## Introduction

Genetic and environmental factors play an important role in the tumorigenesis of cancer. Toxic environmental exposures were estimated to account for 7 to 19% of cancers (1,2). Current knowledge of carcinogens was mainly from the accumulation of animal studies, occupational cohort studies of industrial workers and epidemiological studies of inhabitant exposed to a specific toxicant. The international agency for research on cancer (IARC), National Toxicology Program (NTP) and the US Environmental Protection Agency (EPA) have developed their own classification systems to estimate the carcinogenicity of different chemical, physical and biological substances. The carcinogen lists issued by IARC, NTP and EPA are based on the systematic review of toxicological data by selected experts with specific knowledge (3–5). However, the acquisition of toxicity data is both time- and resource-consuming. For example, the traditional 2-year rodent assays for assessing the carcinogenesis potential of chemicals requires the sacrifice of an extensive amount of animal life (6–9). Given a large number of chemicals in commerce, it is impossible to assess the carcinogenicity of all chemicals based on the traditional assays (10,11). Alternative methods for fast prioritization of chemicals of carcinogenesis concern are therefore of great interest.

Recently, IARC recommended an evidence-based and transparent process for carcinogen identification and characterization focusing on mechanistic information. The US EPA and the NTP also recognized the mechanism-based approach (12). To identify chemicals associated with potential carcinogenesis mechanisms, computational methods provide promising alternatives to experiments and can be utilized as the first-tier prioritization approach. While existing *in silico* methods of structural alerts and quantitative structure-activity relationship (QSAR) models are potential methods for chemical prioritization, the evaluation results provide only a little or no information concerning biological mechanisms. In contrast, *in silico* toxicogenomics methods such as ChemDIS and CTD database could provide mechanism information for carcinogenesis evaluation (13–16). Our recent study demonstrated that the integration of complementary methods of structural alerts, QSARs and *in silico* toxicogenomics methods of genes and Disease Ontology terms associated with developmental and reproductive toxicity effectively improve the performance of chemical prioritization (17). However, there is currently no database of carcinogenesis mechanism-related genes and pathways that is a prerequisite for the development of *in silico* toxicogenomics methods for prioritizing chemicals of carcinogenic concern.

The concept of hallmarks of cancer (HOCs) was introduced by Hanahan and Weinberg to provide a logical framework for understanding the remarkable diversity of neoplastic diseases (18,19). The number of HOCs was expanded and refined during the last two decades. In 2012, the non-profit organization of Getting to Know Cancer launched the Halifax Project based on the framework of hallmarks of cancer. One of the aims of the Halifax Project is to produce a series of overarching reviews written by 703 cancer researchers. The biologically disruptive chemicals ubiquitously found in the environment capable of activating HOCs and the corresponding target genes and relevant pathways were reviewed for a total of 11 HOCs (20). It is therefore desirable to curate the published reviews from Halifax Project consisting of current knowledge of HOC-related mechanism information for further applications to the prioritization of chemicals of concern.

In this study, the unstructured data of genes and pathways from the reviews of Halifax Project were curated and standardized based on ontology terms. An HOC database consisting of 695 genes and 159 pathways denoted as HP genes and HP pathways, respectively, was developed for 11 HOCs. In addition to the HP genes representing selected targets for HOCs, EX genes consisting of additional genes associated with the HP pathways and HP genes were also collected. To demonstrate the usefulness of the curated database, the interacting HP genes, HP pathways and EX genes for chemicals from three carcinogen datasets of the IARC, EPA and NTP were identified based on the targets and enriched pathways generated by ChemDIS analysis. Results show that the number of HOCs involved could be utilized to prioritize chemicals of carcinogenic concern. The curated HOC-related genes and pathways are expected to be useful for characterizing the mechanisms of chemical carcinogens.

## Methods

### Curation of genes and pathways relevant to the hallmarks of cancer

**Figure 1:**
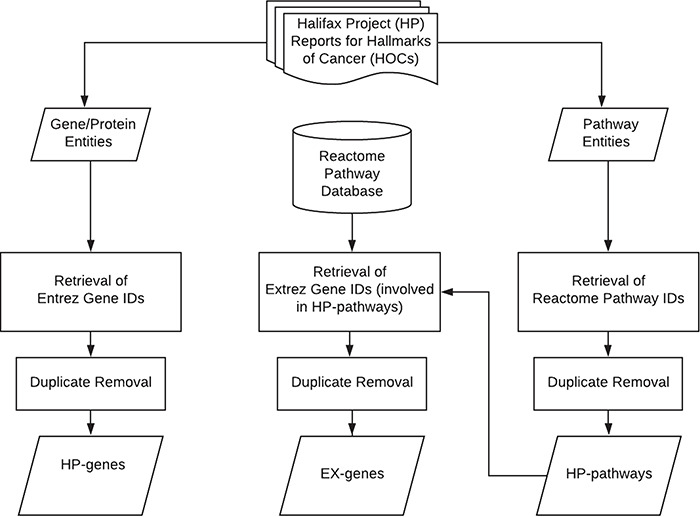
System flow of the curation work for hallmarks of cancer.

The primary aim of this study is to curate HP genes and HP pathways from 11 review articles published in the supplement issue of the journal of *Carcinogenesis* as shown in Table 1 (21–31). Each of the papers represents the unstructured knowledge for a specific hallmark of cancers (HOC). The overview of the data retrieval and curation workflow is shown in Figure 1 and described in the following.

**Table 1:** Review articles for 11 hallmarks of cancer generated by the Halifax Project

Hallmark	Reference
Deregulated metabolism	Robey *et al.*, 2015 (21)
Evasion of anti-growth signaling	Nahta *et al.*, 2015 (22)
Angiogenesis	Hu *et al.*, 2015 (23)
Immune system evasion	Kravchenko *et al.*, 2015 (24)
Resistance to cell death	Narayanan *et al.*, 2015 (25)
Replicative immortality	Carnero *et al.*, 2015 (26)
Sustained proliferative signaling	Engström *et al.*, 2015 (27)
Tissue invasion and metastasis	Ochieng *et al.*, 2015 (28)
Tumor-promoting inflammation	Thompson *et al.*, 2015 (29)
Tumor microenvironment	Casey *et al.*, 2015 (30)
Genome instability	Langie *et al.*, 2015 (31)

The articles were curated and checked by our group. All gene/protein terms and pathway terms mentioned in these articles were manually identified and assigned to the corresponding HOCs. The retrieved gene/protein terms include symbols, names and synonyms. Some of them are symbols or names of a gene/protein family with several members; for example, E2F is a protein family that includes eight family members (E2F1–E2F8) and glutathione peroxidases composed of isoenzymes from different genes (GPX1–GPX8). All gene/protein terms were then submitted to the National Center of Biotechnology Information gene portal (ncbi.nlm.nih.gov/gene) to retrieve the corresponding Entrez gene IDs of humans. For those terms that represent a protein complex that did not return a gene ID, such as PIDDosome, we included all proteins that form that complex. For gene terms that represent gene families, all genes of the same family are retrieved and grouped into the same HOC. The curated gene set consisting of standardized HP genes was named HP gene set (Halifax Project gene set). Duplicate genes were identified and removed for each HOC based on their Entrez gene IDs.

For the retrieval of pathway terms, phrases that are composed of ‘pathway’ or ‘signaling’ were selected, such as ataxia telangiectasia-mutated (ATM) checkpoint kinase 2 (Chk2) pathway or transforming growth factor-beta (TGF-beta) signaling. The pathway terms were then submitted to the Reactome pathway database to identify the corresponding pathways ID (REACTID) that most closely related to the pathway terms (32). The retrieved REACTID of the selected pathways were collected and grouped according to the corresponding HOCs. Duplicate pathway terms were identified and removed for each HOC based on their REACTIDs.

### Expanded gene dataset (EX-genes)

Since the reviews by Halifax Project do not include all relevant genes of an HOC pathway, the HP genes represent only partial information of the HOCs. To obtain more comprehensive gene sets, we expanded the HP genes by appending all genes that participate in the HOC pathways for each HOC. The gene IDs for all REACTIDs were collected from the Reactome database, and duplicates were removed based on the standardized gene IDs. The expanded genes were named EX genes which also included HP genes.

### Reference datasets for validation

A total of three carcinogen lists were extracted from IARC (Monograph 118, https://monographs.iarc.fr/agents-classified-by-the-iarc), NTP (14th Report of Carcinogens, https//ntp.niehs.nih.gov/whatwestudy/assessments/cancer/roc/index.html?utm_source=direct&utm_medium=prod&utm_campaign=ntpgolinks&utm_term=roc) and EPA (dose-response assessment table, Table 1 (chronic) June 18, 2018 https://www.epa.gov/fera/dose-response-assessment-assessing-health-risks-associated-exposure-hazardous-air-pollutants). Chemical names and/or CAS numbers were firstly retrieved from these lists. The PubChem database was subsequently queried using the retrieved names/CAS numbers to identify the corresponding compound IDs (CIDs). Only chemicals with corresponding CIDs and structures were utilized for the validation of curated HOC terms. If a chemical returned more than one CID, the CID number with the synonyms that resemble closely to the chemical will be selected. The CID was kept unique for each HOC.

In the carcinogen list of IARC monograph volumes 1–118, there are a total of 999 agents separated into five groups (Groups 1, 2A, 2B, 3 and 4). After curation, 723 chemical compounds were included for analysis (48 in Group 1, 60 in Group 2A, 229 in Group 2B, 385 in Group 3 and one in Group 4). Fifty-three agents are non-compound agents (such as *Schistosoma haematobium* or betel quid with tobacco). One hundred and sixty-nine agents do not have a corresponding CID. Fifty-four agents have no data on interacting proteins.

The report on carcinogens (RoC) from the NTP website contained agents that listed as ‘known to be a human carcinogen (KHC)’ and ‘reasonably anticipated to be a human carcinogen (RAHC)’. In the 14th RoC, there are 272 agents. Two hundred and twenty compounds are included in the list (34 are KHC and 186 are RAHC). Fourteen agents are non-compound agents (such as UV radiation A or wood dust). Forty-six agents do not have a corresponding CID. Five agents have no data on interacting proteins.

For the EPA carcinogen list, we downloaded the hazardous air pollutants on long-term inhalation and oral exposures. Since the chemicals were classified either based on guidelines published in 1986 or 2005, we regrouped them into five groups: Group A consists of human carcinogens; Group B comprises probable carcinogens; Group C is composed of possible carcinogens; Group D contains unclassifiable chemicals; Group E consists of non-carcinogens (Table S1). The list comprises one hundred and fifty-three chemicals. After removing two chemicals without CIDs, a total of 150 chemicals (13 for Group A, 71 for Group B, 20 for Group C, 44 for Group D and 2 for Group E) were included in our analysis.

### Analysis of affected hallmarks of cancer

For each chemical, its interacting proteins and corresponding enriched pathways were obtained from the toxicogenomics analysis system of ChemDIS. Since the effect on HOCs could be either activation or inactivation, only affected HOCs could be identified by using ChemDIS. In this study, a threshold score of 150 was used for the toxicogenomics analysis and a pathway with an original *P* value<0.05 was considered to be significantly affected. Five criteria were proposed to identify affected HOCs as described in the following.

(a) HP gene:An HOC is considered affected if at least one HP gene of the HOC is interacting with the test chemical.
(b) HP pathway:An HOC is considered affected if at least one HP pathway of the HOC is significantly affected by the test chemical.
(c) EX gene:An HOC is considered affected if at least one EX gene of the HOC is interacting with the test chemical.
(d) HP gene and HP pathway:An HOC is considered affected if at least one HP gene of the HOC is interacting with the test chemical and at least one HP pathway is significantly affected by the test chemical.
(e) EX gene and HP pathway:An HOC is considered affected if at least one EX gene of the HOC is interacting with the test chemical and at least one HP pathway is significantly affected by the test chemical.

### Statistical analysis

Statistical analyses were performed using RStudio setting the significance level *α* = 0.05 (33). The numbers of affected HOCs for chemicals of different toxicity groups defined by IARC and EPA were compared using Dunnett T3 test, which is a modified Tukey–Kramer pairwise multiple comparison *post hoc* tests adjusted for unequal variances and unequal sample sizes (34,35). The number of affected HOC for chemicals in NTP was compared using Student’s *t* test. It was used to validate if our predictive system is able to differentiate the chemicals of each toxicity group using the affected HOCs.

## Results and discussion

### Curation of HP genes and HP pathway

The HP genes and HP pathways related to HOCs were manually curated from the review articles generated by Halifax Project. The curated database consists of 695 HP genes and 159 HP pathways involved with the 11 HOCs. After the expansion of genes according to the involved pathways, the total number of EX genes is 3199. Table 2 shows the numbers of genes (HP genes and EX genes) and pathways (HP pathways) associated with each HOC. Some of the HOCs might be poorly characterized. For example, there are only 4 and 10 HP genes for genome instability and inflammation. Also, the numbers of HP pathways for the HOCs of micro-environment and inflammation are only 3 and 2. The HOCs with a small number of HP genes and HP pathways might result in lower sensitivity for the detection of affected HOCs based on the proposed strategies as mentioned in Method. The curated HOC database is available in the  Supplementary Table S2.

**Table 2:** The number of curated genes (HP genes), pathways (HP pathways) and expanded genes (EX genes)

Hallmark of cancer (HOC)	HP gene	HP pathway	EX gene
Evasion of anti-growth signaling	160	19	427
Replicative immortality	56	17	429
Sustained proliferation	75	14	246
Genome instability	4	12	292
Resistance to cell death	102	15	235
Immune evasion	26	17	259
Invasion and metastasis	101	7	232
Micro-environment	43	3	109
Angiogenesis	26	10	242
Metabolism	126	25	644
Inflammation	10	2	84

**Figure 2:**
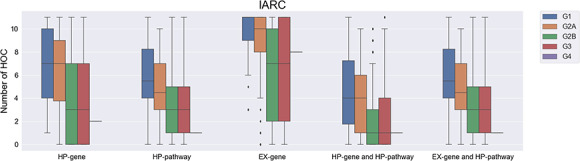
The number of affected hallmarks of cancer (HOCs) for different groups of IARC chemicals. IARC, international agency for research on cancer; G1, IARC Group 1 chemicals; G2A, IARC Group 2A chemicals; G2B, IARC Group 2B chemicals; G3, IARC Group 3 chemicals; G4, IARC Group 4 chemicals.

### Application of curated HOC data to the prioritization of chemicals of carcinogenic concern

To demonstrate the usefulness of the curated HOC database, three carcinogen lists of IARC, NTP and EPA were analyzed based on the analysis results of interacting genes and enriched pathways by the *in silico* toxicogenomics system ChemDIS. First, the interacting HP genes, EX genesand HP pathways were identified using the ChemDIS system. Subsequently, the activation of HOCs was calculated based on five criteria of HP gene, EX gene, HP pathway, the combination of HP gene and HP pathway and the combination of EX gene and HP pathway. Finally, statistical significance was calculated using the Dunnett T3 test.

For the IARC list, we found an increasing trend in the number of affected HOCs when the corresponding carcinogenicity category of a compound increases (Figure 2). Based on different criteria (HP gene, EX gene and HP pathway), the number of affected HOCs involved in Group 1 and Group 2A chemicals is significantly higher (*P* value ≤0.05) than those involved by group 2B and Group 3/Group 4. Such difference is also seen when genes (HP genes or EX genes) and HP pathways are considered together (i.e. the combination of HP gene and HP pathway, and the combination of EX gene and HP pathway). Altogether, the results suggest that chemicals interacting with more HP genes/EX genes/HP pathways are more likely to be a carcinogen.

**Figure 3:**
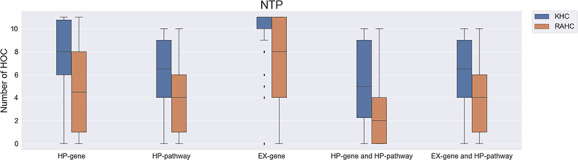
The number of affected hallmarks of cancer (HOCs) for different groups of National Toxicology Program chemicals. NTP, National Toxicology Program; KHC, known to be a human carcinogen; RAHC, reasonably anticipated to be a human carcinogen.

Similar results were also found in the analysis of the NTP carcinogen list (Figure 3), where chemicals with a higher number of interacting proteins involved with HOCs are more likely to be carcinogenic (*P* value ≤0.005).

**Figure 4:**
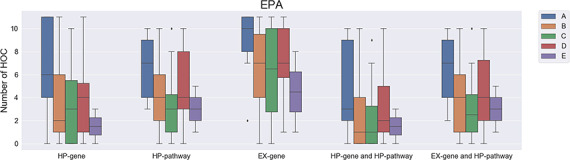
The number of affected hallmarks of cancer (HOCs) for different groups of Environmental Protection Agency chemicals. EPA, Environmental Protection Agency; A, Group A chemicals after regroup chemicals classes that previously classified based on the EPA guidelines published in 1986 or 2005; B, Group B; C, Group C; D, Group D; E, Group E.

For the EPA list, similar results were also obtained by the comparison of chemicals in Group A, Group B and Group C, but not chemicals in Group D and Group E (Figure 4). When using methods such as HP gene, EX gene, HP pathway, the combination of HP gene and HP pathway and the combination of EX gene and HP pathway to determine the affected HOCs, the number of affected HOCs involved in Group A is significantly higher than those in Group B and Group C. Although a higher number of affected HOCs were found in Group B when compared to Group C, it is not statistically significant with a *P* value ≥0.872. Also, the numbers of affected HOCs in Group D and Group E are not statistically different from Group A chemicals with a *P* value ≥0.222. It is easy to understand why the number of affected HOCs for chemicals in Group D is not statistically significant since the chemicals in Group D still have no sufficient information to make a conclusion and might be carcinogens. However, the number of affected HOCs in Group D is lower than that in Group A, and thus the chemicals in the latter group still can be separated out with a proper cutoff value. Since the system for classifying the carcinogenicity of agents made a major shift in 2005, the combination of two different classification systems might perturb the analysis. Besides, the mean numbers of affected HOCs in Group D are lower than those in Group A but higher than those in Group B and Group C. Please note that the mean number of affected HOCs in Group D is subject to change once the chemicals are reclassified based on new evidence.

The criteria applied for the classification of the carcinogens are different among the agencies; thus, duplicated and/or inconsistently classified chemicals are common among the datasets of EPA, NTP and IARC. There are 28 chemicals that are duplicated or inconsistently classified, 13 of them are listed in all three of the carcinogen lists and 15 of them are listed in at least two out of three of the carcinogen lists (Table S3). The carcinogenicity classification of 14 of the chemicals is inconsistent in different carcinogen lists, listed as carcinogen (known human carcinogen in NTP, grade 1 in IARC and/or Class A in EPA) in some of the lists but not in other. The details of the inconsistency are listed in Table S3 and Table S4. The chemicals in the EPA carcinogen lists have the highest inconsistency rate, with 8% (*n* = 12) of them inconsistently classified in the other two lists, followed by 5.9% (*n* = 13) in NTP and 1.7% (*n* = 12) in IARC. The removal of the small number of inconsistently or duplicated chemicals did not affect the conclusions.

Altogether, the analysis results for the three carcinogen lists show that chemicals with more affected HOCs are more likely to be more carcinogenic. The integration of the HOC database and ChemDIS analysis is useful. Since the analysis of ChemDIS is based on known and predicted interacting proteins, the assessment results of poorly characterized chemicals with an incomplete profile of interacting proteins could be unreliable. The affected HOCs could also be identified by using experimental data such as differentially expressed genes derived from the transcriptomics study to further improve the prioritization method.

## Conclusion

This study presents a novel database of HOC-relevant genes and pathways curated from the review articles published by the Halifax Project (HP). The database enables the identification of affected HOCs associated with a chemical once we know the interacting genes and pathways of the chemical. Besides, by using the publicly available Reactome pathway database, we further expanded the HOC-relevant gene set that was not mentioned in the HP articles but is likely to work alongside the chemicals indirectly. This step is essential since some of the Halifax Project reports focus on the discussion of HOCs mechanisms rather than relevant genes. The inclusion of the extra genes associated with HOC relevant pathways is therefore considered a vital add-on to the analysis of affected HOCs.

Since the number of affected HOCs reflects how extensive a chemical involved in the cancer-related pathway of a cell, we incorporated the analysis results of interacting genes and enriched pathways of chemicals from ChemDIS to develop an *in silico* prioritization method for chemicals of carcinogenic concern. Five criteria were proposed to identify affected HOCs, and three carcinogen lists of IARC, NTP and EPA were utilized to validate the proposed prioritization method. Our results show that chemicals with more affected HOCs are more likely to be more carcinogenic and the HOC database is useful.

This important finding demonstrated that the HOC information can provide insights into the mechanisms of how a carcinogen transforming cell. For example, aristolochic acid (AA) derived from the *Aristolochia* spp. is a genotoxic mutagen that formed DNA adducts after metabolic activation (36). It is grouped as G1 in IARC monograph volumes 1–118. Using the OECD QSAR toolbox and the VEGA QSAR application, AA is labeled as a carcinogen (37,38). With the information of HOC, we can disclose that AA interacts directly or indirectly with the genes of evasion of anti-growth signaling, replicative immortality, sustained proliferation, cell death, immune evasion, invasion and metastasis, microenvironment, angiogenesis and metabolism. These are the possible mechanisms that AA transformed during the process of carcinogenesis. The information of HOC can also be used as additional methods that work along with the QSAR methods to predict carcinogenicity of chemicals. Silica dust, crystalline in the form of quartz or cristobalite, is listed as a G1 carcinogen in the IARC monograph (39). Silica dust did not raise carcinogen alert when evaluated using the OECD QSAR toolbox and the VEGA QSAR application. However, the HOC database shows that silica dust interacts with all of the 11 HOC. Thus, the knowledge of the HOC database can be important complementary information besides QSAR analysis.

This database can be a valuable tool for toxicologists and researchers that are interested in understanding the mechanistic pathways that transform a normal cell into a cancer cell by a chemical. Given test chemicals, the potentially affected HOCs can be easily identified the chemicals can be prioritized for their carcinogenic potential based on the *in silico* method shown in this study. While the affected HOCs of a chemical identified by the proposed method combining HOC database and ChemDIS analysis is useful for prioritizing chemicals of carcinogenic concern, the activation or inactivation effect on the HOCs can be further studied to provide insights into the molecular pathways. Future works could be the incorporation of the differentially expressed genes derived from toxicogenomics studies for improving the identification of affected HOCs and determination of the action of HOCs. The curated HOC database is expected to be useful for identifying chemicals of carcinogenic concern and could be further integrated with other evidence for chemical prioritization based on the weight-of-evidence principle.

## Supplementary Material

Suppl_data_baaa045
