# Supplementary material for: Curation of cancer hallmark-based genes and pathways for in silico characterization of chemical carcinogenesis
Source: Database (Oxford). 2020 Jun 15;2020:baaa045. doi: 10.1093/database/baaa045 (PMC7294774; doi:10.1093/database/baaa045)
Supplement: Suppl_data_baaa045 [file suppl_data_baaa045.zip › Halifax-Curation.Table S1.docx]

Table S1. Regroup of the chemicals classes that previously classified based on the EPA guidelines published in 1986 or 2005.

| Regroup | EPA WOE 1986 classification | EPA WOE 2005 classification |
| --- | --- | --- |
| Group A | A = human carcinogen | CH = carcinogenic to humans |
| Group B | B1 = probable carcinogen, limited human evidence  B2 = probable carcinogen, sufficient evidence in animals | LH = likely to be carcinogenic |
| Group C | C = possible human carcinogen | SE = suggestive evidence of carcinogenic potential |
| Group D | D = not classifiable | InI = inadequate information to assess carcinogenic potential |
| Group E | E = evidence of noncarcinogenicity | NH = not likely to be carcinogenic |
