# Supplementary material for: Curation of cancer hallmark-based genes and pathways for in silico characterization of chemical carcinogenesis
Source: Database (Oxford). 2020 Jun 15;2020:baaa045. doi: 10.1093/database/baaa045 (PMC7294774; doi:10.1093/database/baaa045)
Supplement: Suppl_data_baaa045 [file suppl_data_baaa045.zip › Halifax-curation.Table S3.docx]

Table S3. Duplicated or Inconsistent Chemicals Across the EPA, IARC, and NTP carcinogen lists.

| CID_No | EPA-NTP | EPA-IARC | IARC-NTP |
| --- | --- | --- | --- |
| CID000000712 | Inconsistent in Carcinogenicity | Inconsistent in Carcinogenicity | Duplicate |
| CID000000727 | Duplicate | Inconsistent in Carcinogenicity | Inconsistent in Carcinogenicity |
| CID000000934 | Inconsistent in Carcinogenicity | Inconsistent in Carcinogenicity | Duplicate |
| CID000000992 | Duplicate | Inconsistent in Carcinogenicity | Inconsistent in Carcinogenicity |
| CID000000996 |  | Duplicate |  |
| CID000001775 |  |  | Duplicate |
| CID000002336 | Inconsistent in Carcinogenicity | Duplicate | Inconsistent in Carcinogenicity |
| CID000002514 | Inconsistent in Carcinogenicity |  |  |
| CID000004053 |  |  | Duplicate |
| CID000004754 |  |  | Inconsistent in Carcinogenicity |
| CID000004915 |  |  | Duplicate |
| CID000006338 | Duplicate | Duplicate | Duplicate |
| CID000006564 |  | Inconsistent in Carcinogenicity |  |
| CID000006658 |  | Duplicate |  |
| CID000007000 |  |  | Duplicate |
| CID000007070 | Duplicate | Duplicate | Duplicate |
| CID000007242 | Inconsistent in Carcinogenicity | Inconsistent in Carcinogenicity | Duplicate |
| CID000007251 |  |  | Duplicate |
| CID000007501 |  |  | Duplicate |
| CID000007543 | Duplicate | Inconsistent in Carcinogenicity | Inconsistent in Carcinogenicity |
| CID000007577 |  |  | Duplicate |
| CID000008461 |  | Duplicate |  |
| CID000011443 |  |  | Duplicate |
| CID000014012 | Duplicate |  |  |
| CID000015625 | Inconsistent in Carcinogenicity | Inconsistent in Carcinogenicity | Duplicate |
| CID000031369 | Duplicate | Duplicate | Duplicate |
| CID000036231 | Duplicate | Inconsistent in Carcinogenicity | Inconsistent in Carcinogenicity |
| CID000107649 | Inconsistent in Carcinogenicity | Inconsistent in Carcinogenicity | Duplicate |
