# Supplementary material for: Curation of cancer hallmark-based genes and pathways for in silico characterization of chemical carcinogenesis
Source: Database (Oxford). 2020 Jun 15;2020:baaa045. doi: 10.1093/database/baaa045 (PMC7294774; doi:10.1093/database/baaa045)
Supplement: Suppl_data_baaa045 [file suppl_data_baaa045.zip › Halifax-curation.Table S4.docx]

Table S4. Number of Duplicated or Inconsistent Chemicals Across the EPA, IARC, and NTP carcinogen lists.

| Relationship | Inconsistent in Carcinogenicity | Duplicate |
| --- | --- | --- |
| EPA-NTP | 7 | 8 |
| EPA-IARC | 10 | 7 |
| IARC-NTP | 7 | 15 |
